# Metabolic engineering of *Corynebacterium glutamicum* for enhanced production of 5-aminovaleric acid

**DOI:** 10.1186/s12934-016-0566-8

**Published:** 2016-10-07

**Authors:** Jae Ho Shin, Seok Hyun Park, Young Hoon Oh, Jae Woong Choi, Moon Hee Lee, Jae Sung Cho, Ki Jun Jeong, Jeong Chan Joo, James Yu, Si Jae Park, Sang Yup Lee

**Affiliations:** 1 Department of Chemical and Biomolecular Engineering (BK21 Plus program), Institute for the BioCentury, Center for Systems and Synthetic Biotechnology, KAIST, 291 Daehak-ro, Yuseong-gu, Daejeon, 34141 Republic of Korea; 2 Metabolic Engineering National Research Laboratory and BioProcess Engineering Research Center, KAIST, 291 Daehak-ro, Yuseong-gu, Daejeon, 34141 Republic of Korea; 3 Bioinformatics Research Center, KAIST, 291 Daehak-ro, Yuseong-gu, Daejeon, 34141 Republic of Korea; 4 Division of Convergence Chemistry, Center for Bio-based Chemistry, Korea Research Institute of Chemical Technology, P.O. Box 107, 141 Gajeong-ro, Yuseong-gu, Daejeon, 34602 Republic of Korea; 5 Department of Environmental Engineering and Energy, Myongji University, 116 Myongji-ro, Cheoin-gu, Yongin, Gyeonggido 17058 Republic of Korea

**Keywords:** 5-Aminovaleric acid, *Corynebacterium glutamicum*, l-Lysine, Metabolic engineering, Glutaric acid

## Abstract

**Background:**

5-Aminovaleric acid (5AVA) is an important five-carbon platform chemical that can be used for the synthesis of polymers and other chemicals of industrial interest. Enzymatic conversion of l-lysine to 5AVA has been achieved by employing lysine 2-monooxygenase encoded by the *davB* gene and 5-aminovaleramidase encoded by the *davA* gene. Additionally, a recombinant *Escherichia coli* strain expressing the *davB* and *davA* genes has been developed for bioconversion of l-lysine to 5AVA. To use glucose and xylose derived from lignocellulosic biomass as substrates, rather than l-lysine as a substrate, we previously examined direct fermentative production of 5AVA from glucose by metabolically engineered *E. coli* strains. However, the yield and productivity of 5AVA achieved by recombinant *E. coli* strains remain very low. Thus, *Corynebacterium glutamicum*, a highly efficient l-lysine producing microorganism, should be useful in the development of direct fermentative production of 5AVA using l-lysine as a precursor for 5AVA. Here, we report the development of metabolically engineered *C. glutamicum* strains for enhanced fermentative production of 5AVA from glucose.

**Results:**

Various expression vectors containing different promoters and origins of replication were examined for optimal expression of *Pseudomonas putida davB* and *davA* genes encoding lysine 2-monooxygenase and delta-aminovaleramidase, respectively. Among them, expression of the *C. glutamicum* codon-optimized *davA* gene fused with His_6_-Tag at its N-Terminal and the *davB* gene as an operon under a strong synthetic H_36_ promoter (plasmid p36davAB3) in *C. glutamicum* enabled the most efficient production of 5AVA. Flask culture and fed-batch culture of this strain produced 6.9 and 19.7 g/L (together with 11.9 g/L glutaric acid as major byproduct) of 5AVA, respectively. Homology modeling suggested that endogenous gamma-aminobutyrate aminotransferase encoded by the *gabT* gene might be responsible for the conversion of 5AVA to glutaric acid in recombinant *C. glutamicum*. Fed-batch culture of a *C. glutamicum gabT* mutant-harboring p36davAB3 produced 33.1 g/L 5AVA with much reduced (2.0 g/L) production of glutaric acid.

**Conclusions:**

*Corynebacterium glutamicum* was successfully engineered to produce 5AVA from glucose by optimizing the expression of two key enzymes, lysine 2-monooxygenase and delta-aminovaleramidase. In addition, production of glutaric acid, a major byproduct, was significantly reduced by employing *C. glutamicum gabT* mutant as a host strain. The metabolically engineered *C. glutamicum* strains developed in this study should be useful for enhanced fermentative production of the novel C5 platform chemical 5AVA from renewable resources.

**Electronic supplementary material:**

The online version of this article (doi:10.1186/s12934-016-0566-8) contains supplementary material, which is available to authorized users.

## Background

As a result of increasing pressure on the environment, bio-based production of chemicals, fuels, and materials from renewable non-food biomasses has been attracting much attention [1]. To make such bio-based processes competitive, microorganisms have been metabolically engineered for production of fuels [2–4], amino acids [5–9], polymers [10–12], and other chemicals of industrial importance [13–15]. It is expected that more chemicals and materials of petrochemical origin will be produced through bio-based route employing microorganisms developed by systems metabolic engineering [16, 17].

A non-proteinogenic *ω*-amino acid, 5-aminovaleric acid (5AVA), has attracted attention as a five carbon (C5) platform chemical because of its potential in polymer synthesis [18–21]. 5AVA can be used to produce *δ*-valerolactam (2-piperidone) via intramolecular dehydrative cyclization and can be further processed for synthesis of bio-based nylons, such as nylon-5 and nylon-6,5 [18, 20]. Enzymatic conversion of l-lysine to 5AVA has been achieved by employing lysine 2-monooxygenase (E.C. 1.13.12.2, encoded by the *davB* gene) and 5-aminovaleramidase (E.C. 3.5.1.30, encoded by the *davA* gene) [19]. We recently reported the development of a whole-cell bioconversion process for conversion of l-lysine to 5AVA by employing recombinant *Escherichia coli* strains expressing lysine 2-monooxygenase and 5-aminovaleramidase as whole cell biocatalysts [20, 21]. However, it is obviously desirable to use glucose derived from non-food lignocellulosic biomass as a substrate rather than l-lysine [1]. There have been reports on the development of metabolically engineered microorganisms for the production of C3 and C4 *ω*-amino acids, such as β-alanine [22] and γ-aminobutyrate [23], from glucose. We and others also examined the possibility of producing the C5 *ω*-amino acid 5AVA by metabolic engineering of *E. coli*, but the yield and productivity of 5AVA remain very low [18, 20]. Thus, it is necessary to develop a new strategy for more efficient production of 5AVA.

*Corynebacterium glutamicum* is an organism widely used for the production of amino acids, proteins, monomers for plastic materials, and compounds for cosmetics [24, 25]. Additionally, *C. glutamicum* has been successfully engineered to produce a different C5-platform chemical, cadaverine (1,5-pentanediamine), and was shown to be a promising host for producing this chemical using different carbon sources, such as glucose and xylose [26–30]. Because *C. glutamicum* strains capable of producing l-lysine at very high levels have already been commercialized, we decided to exploit *C. glutamicum* as a host strain for the production of 5AVA.

In this study, we designed and introduced a synthetic pathway for the production of 5AVA into an l-lysine-overproducing *C*. *glutamicum* strain. The synthetic pathway consists of two key enzymes, lysine 2-monooxygenase encoded by the *davB* gene and 5-aminovaleramidase encoded by the *davA* gene, responsible for the conversion of l-lysine to 5AVA. Various expression systems including vectors and promoters were examined for the most efficient production of 5AVA in *C*. *glutamicum*. A reaction responsible for unexpected formation of glutaric acid as a major byproduct was identified and subsequently deleted. The final engineered *C*. *glutamicum* strain was used for enhanced production of 5AVA by fermentation with much reduced glutaric acid.

## Results and discussion

### Construction of the 5AVA synthesis pathway in *C*. *glutamicum* by expressing the *davAB* genes under the control of the *tac* promoter

Although 5AVA exists as an intermediate in amino acid degradation pathways in Pseudomonads, metabolic engineering for enhanced production of 5AVA requires strong metabolic flux from the chosen intermediate metabolite toward 5AVA as indicated by many successful examples of systems metabolic engineering [16, 17]. Recombinant *E. coli* strains employing the *davAB* genes from the l-lysine catabolic pathway of Pseudomonads were previously shown to produce 5AVA, although at low yield and productivity [18, 21]. Because *E. coli* strains have a relatively low capacity to provide l-lysine as a precursor for 5AVA, high-level production of 5AVA could not be achieved in recombinant *E. coli* strains even though the l-lysine catabolic pathway via 5-aminovaleramide provides the shortest route from l-lysine to 5AVA [18, 21]. Thus, *C. glutamicum*, the well-known, highly efficient l-lysine producing bacterium, was selected as a host strain for 5AVA production in this study to provide strong metabolic flux from glucose to l-lysine, the direct precursor of 5AVA [5, 6]. This is strategically advantageous for producing compounds using l-lysine as a direct precursor.

**Fig. 1:**
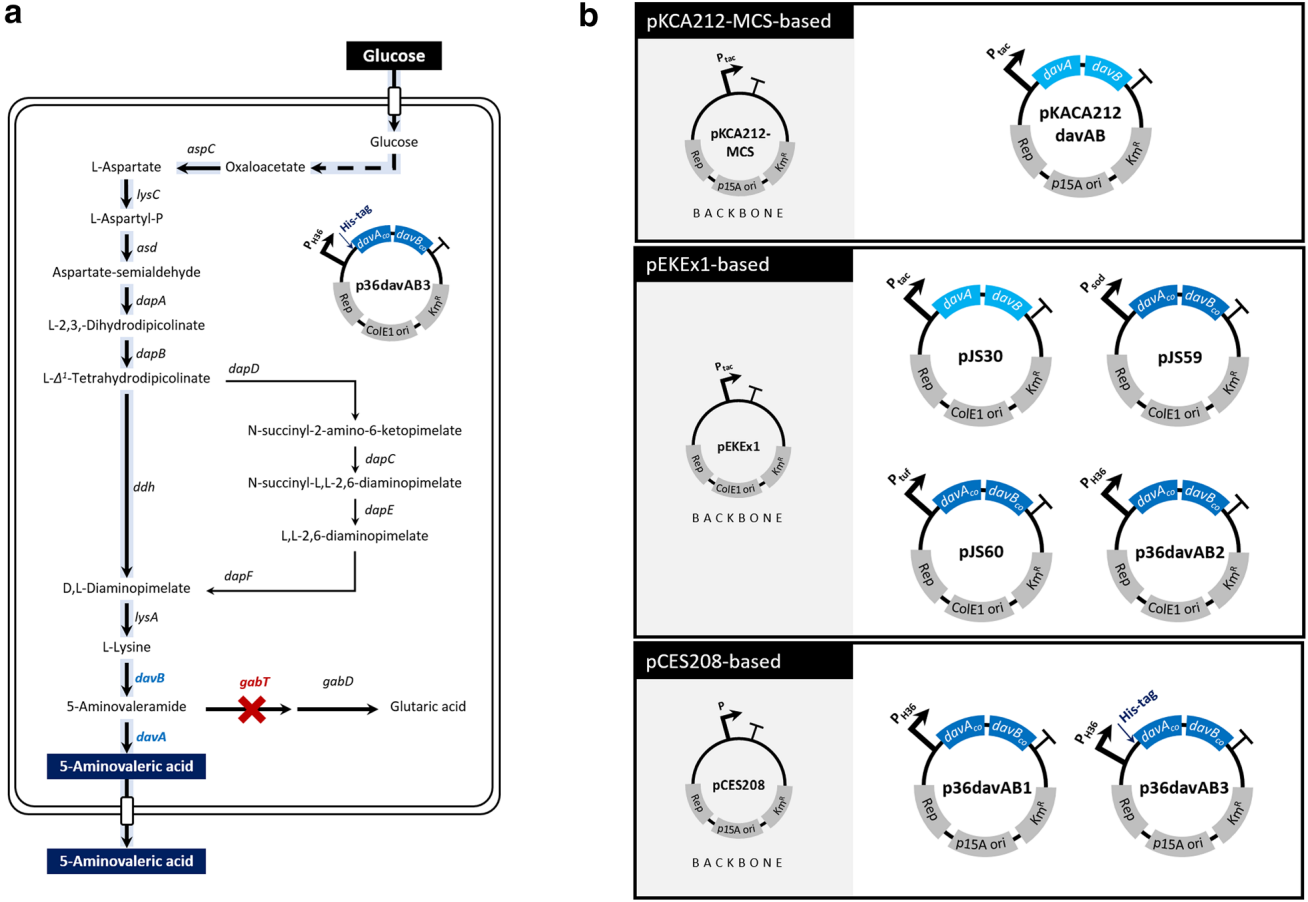
Metabolic engineering strategies for 5AVA production using *C*. *glutamicum*. Heterologous expression of the *P. putida davB* gene (encoding l-lysine 2-monooxygenase) and the *davA* gene (encoding delta-aminovaleramidase) results in conversion of l-lysine into 5AVA. *5AVA5*, 5-aminovalerate; *ASP5* l-aspartate; *ASP-P* aspartyl phosphate; *ASP-SA* aspartate semialdehyde; *LYS* l-lysine

**Fig. 2:**
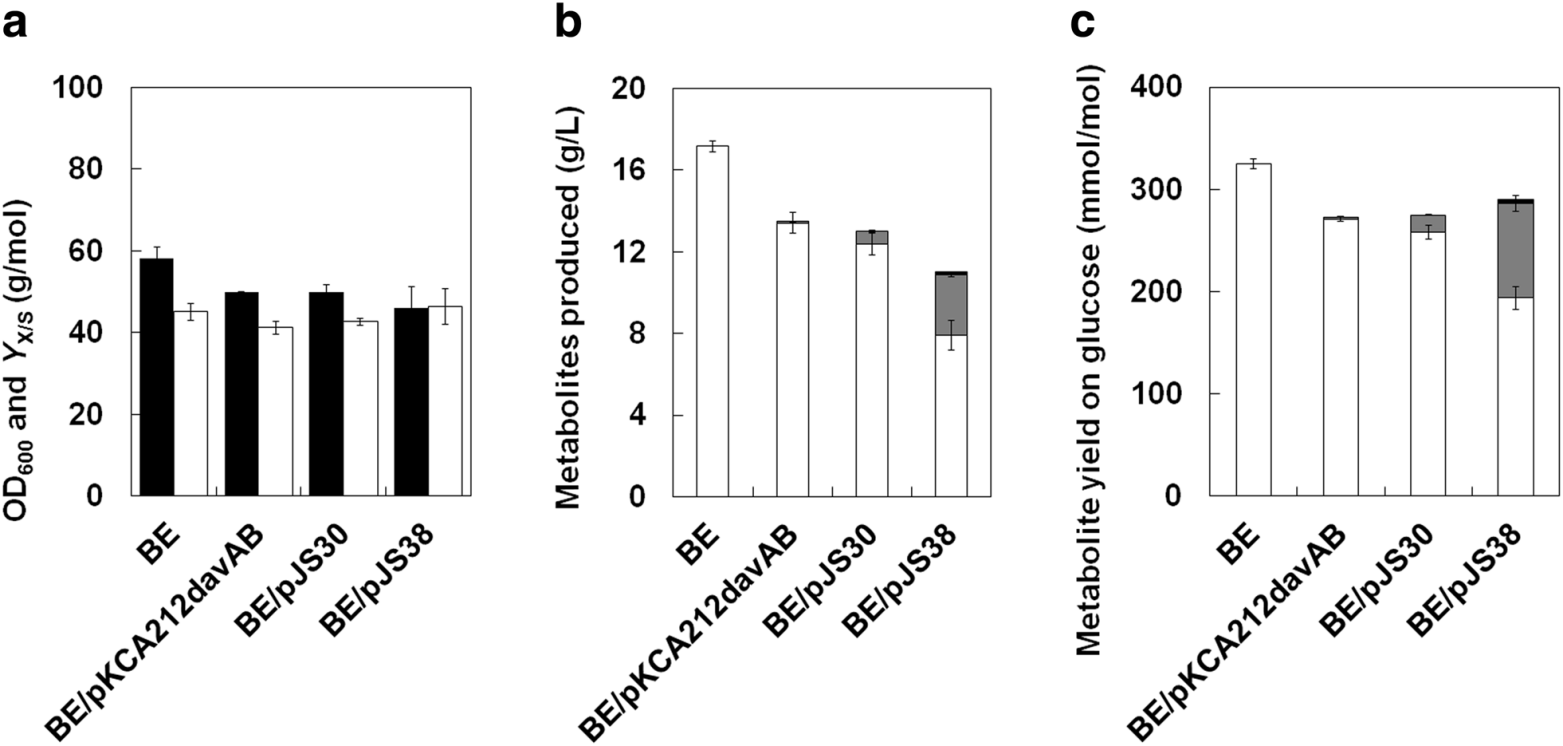
Growth and production characteristics of the *C*. *glutamicum* BE strain harboring the pKCA212davAB, pJS30, or pJS38 vectors after 44 h of shake-flask cultivation (*n* = 3, *error bars* = SD). The *C*. *glutamicum* BE strain containing no plasmids was used as a non-engineered control. **a** The final OD_600_ at the end of cultivation is shown for all strains tested, and an experimentally determined correlation factor (0.28) was used to determine the biomass yield (*Y*_X/S_). **b** The production characteristics include the final titers for l-lysine (*light-grey bars*), 5AVA (*dark-grey bars*), and glutaric acid (*black bars*). **c** The molar yields from glucose for l-lysine (*white bars*), 5AVA (*grey bars*), and glutaric acid (*black bars*) are also shown. When appropriate, gene expression was induced by addition of IPTG at a final concentration of 0.5 mM when the growth reached an OD_600_ of 0.5–0.6

To extend the metabolic flux from glucose toward 5AVA beyond l-lysine (Fig. 1a), *P*. *putida* ATCC 12633 *davAB* genes [20, 21] were cloned in two different *E*. *coli*-*C*. *glutamicum* shuttle vectors (Additional file 5: Table S1; Fig. 1b) and expressed in *C*. *glutamicum* BE strain (KCTC 12390BP). In plasmids pKCA212davAB and pJS30 (Fig. 1b), the *davA* and *davB* genes were organized in an operon and expressed under the control of the *tac* promoter, with an additional *lacUV5* ribosome-binding site (tttcacacaggaaaca) for the *davB* gene residing between the coding sequences of the two genes. Plasmid pKCA212davAB was constructed based on an in-house shuttle vector, pKCA212-MCS, whereas pJS30 was derived from pEKEx1 [31]. Plasmid pEKEx1 contains a pBL1 origin of replication, having ~30 copy numbers per cell in *C*. *glutamicum* [32], and pKCA212-MCS contains a pCC1 origin of replication, also having ~30 to ~40 copies per cell [33]. It was found that *C*. *glutamicum* BE could produce 17.2 g/L l-lysine (yield of 325 mmol/mol glucose) in shake-flask cultivation in 44 h (Fig. 2). It was found through HPLC analysis of metabolites excreted into culture medium that expression of the *davAB* genes in *C*. *glutamicum* BE resulted in conversion of l-lysine to 5AVA, whereas the non-engineered strain did not convert any l-lysine into 5AVA (Fig. 2).

Slightly decreased l-lysine production was observed in recombinant *C*. *glutamicum* BE strains expressing the *davAB* genes, although l-lysine was still the major product in both engineered strains tested. Expression of the *davAB* genes using the shuttle vector pEKEx1 (pJS30) was more effective at producing 5AVA than using pKCA212-MCS (pKCA212davAB) (Fig. 2). The *C*. *glutamicum* BE strain harboring pKCA212davAB produced 13.4 g/L l-lysine (yield of 271.2 mmol/mol) and 58 mg/L 5AVA (yield of 1.5 mmol/mol), whereas the *C*. *glutamicum* BE strain harboring pJS30 produced 12.4 g/L l-lysine (yield of 258.2 mmol/mol) and 641 mg/L 5AVA (yield of 16.7 mmol/mol) from glucose. These results demonstrate that heterologous expression of the *davAB* genes from gram-negative *P*. *putida* correctly functioned to produce 5AVA from glucose using l-lysine as a 5AVA precursor in gram-positive *C*. *glutamicum*.

Although the *C*. *glutamicum* BE strain harboring pKCA212davAB or pJS30 successfully produced 5AVA from glucose, most of l-lysine was not converted into 5AVA, which suggests that metabolic flux from l-lysine to 5AVA was still quite weak as a result of inefficient expression of the *davAB* genes. Thus, we investigated whether 5AVA production could be enhanced by employing *C*. *glutamicum* codon-optimized *davAB* genes. The *C*. *glutamicum* BE strain harboring pJS38, which expresses *C*. *glutamicum* codon-optimized *davAB* genes, produced 3.0 g/L 5AVA in flask cultivation, which represented an increase of almost 370 % over that produced by the *C*. *glutamicum* BE strain harboring pJS30 (Fig. 2). However, 7.9 g/L l-lysine still remained in the culture medium of *C*. *glutamicum* BE strain (pJS38). The yield for l-lysine obtained by *C*. *glutamicum* BE (pJS38) was 193.9 mmol/mol glucose, whereas that for 5AVA was 92.3 mmol/mol glucose.

Notably, 0.14 mg/L glutaric acid was detected in the culture medium of *C*. *glutamicum* BE (pJS38), even though 5AVA aminotransferase and glutarate-semialdehyde dehydrogenase (encoded by *davT* and *davD*, respectively), which are the key enzymes for further conversion of 5AVA into glutaric acid using *α*-ketoglutarate as an amine acceptor [18, 21], were not expressed in this strain. This result strongly suggests that endogenous enzymes homologous to 5AVA aminotransferase and glutarate-semialdehyde dehydrogenase might be involved in further conversion of 5AVA into glutaric acid in *C. glutamicum*.

### Examination of 5AVA production by engineered *C*. *glutamicum* strain expressing the *davAB* genes under the control of the *tac* promoter in fed-batch fermentation

**Fig. 3:**
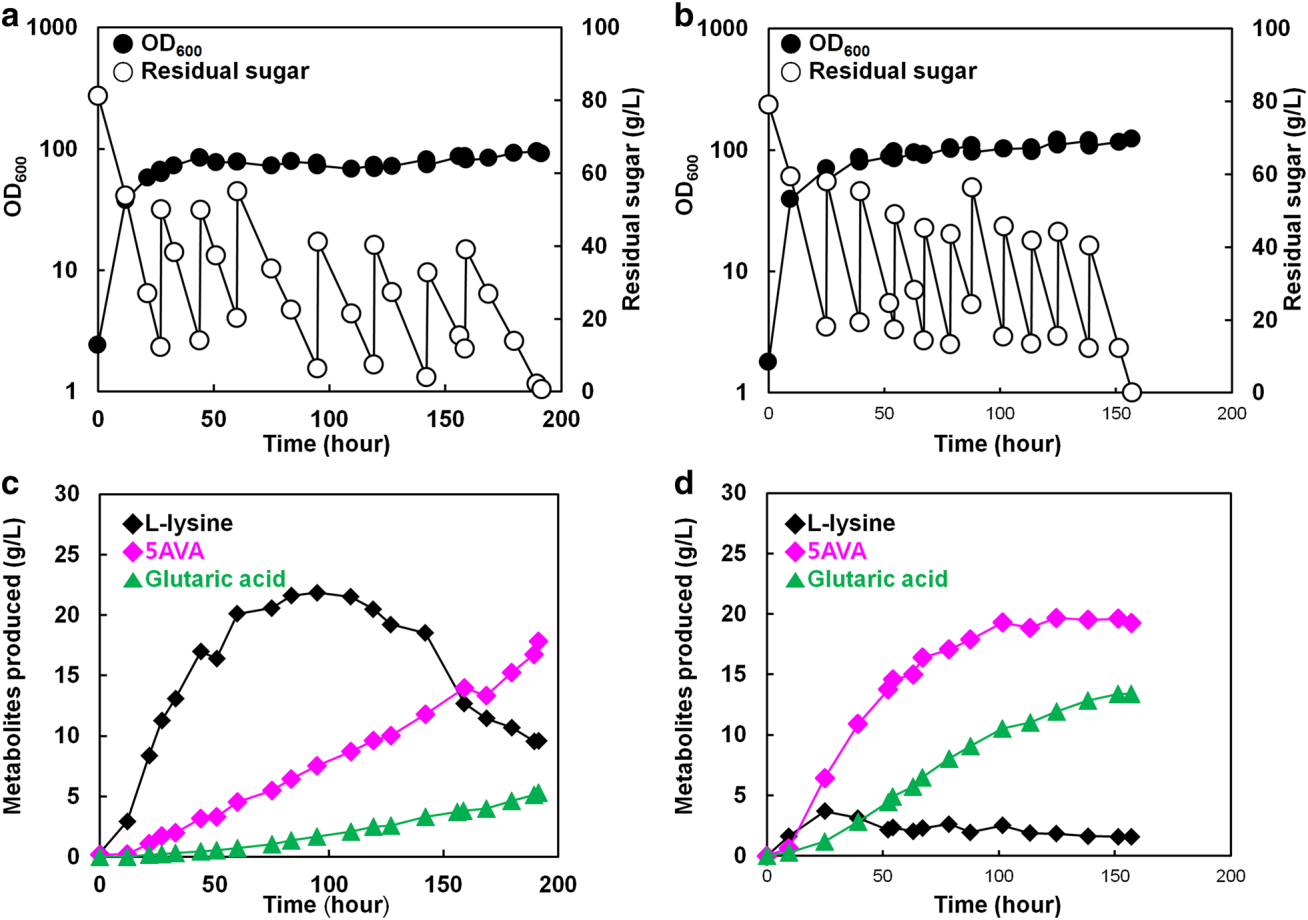
Production of 5AVA from glucose by fed-batch cultures of (**a**, **c**) *C*. *glutamicum* BE (pJS38) and (**b**, **d**) *C*. *glutamicum* BE (p36davAB3). Characteristics of the fed-batch cultivation profile, including growth (*filled circles*, OD_600_), residual sugar (*empty circles*; g/L), l-lysine (*filled diamonds*), 5AVA (*magenta diamonds*), and glutaric acid (*green triangles*) production titers, are plotted against the cultivation time

We then investigated the capability of *C*. *glutamicum* BE (pJS38) to produce 5AVA by fed-batch fermentation in a 5-L fermentor (Fig. 3a, c) to examine its potential for large-scale production of 5AVA. During fed-batch cultivation of *C*. *glutamicum* BE (pJS38), l-lysine concentration reached 21.8 g/L after 94 h of cultivation, and then decreased beyond this point. *C*. *glutamicum* BE (pJS38) produced 17.8 g/L 5AVA in 191 h, with an overall yield and productivity of 0.07 g/g (107.3 mmol/mol) and 0.09 g/L/h, respectively (Fig. 3c). The total input of glucose was 790 g (4.4 mol), and the total amount of produced 5AVA was 55.3 g (471 mmol), with the final volume of 3.1 L. The maximum specific growth rate was 0.23 h^−1^, and the observed maximum specific productivity was 9.2 mg/g/h. Initial increases in l-lysine levels followed by a decrease in titer along with constant production of 5AVA indicated that l-lysine-production flux might be strong initially before slowing down in the latter half of cultivation. The maximum l-lysine-specific productivity was 56.7 mg/g/h initially, but gradually decreased to zero. The amount of the major byproduct, glutaric acid, also increased steadily throughout the entire cultivation period until the concentration reached 5.3 g/L at the end of cultivation (Fig. 3c).

The maximum OD_600_ reached was 95.4, corresponding to the measured dry cell weight concentration of 24.5 g/L, after 189 h of fermentation (Fig. 3a). These results demonstrated that *C*. *glutamicum* BE (pJS38) was able to successfully produce 5AVA from renewable resources in a laboratory-scale bioreactor. However, conversion of l-lysine toward 5AVA needs to be further enhanced through stronger expression of the *davAB* genes.

### Construction of engineered *C. glutamicum* strain expressing the *davAB* genes under the control of constitutive promoters to improve 5AVA production

**Fig. 4:**
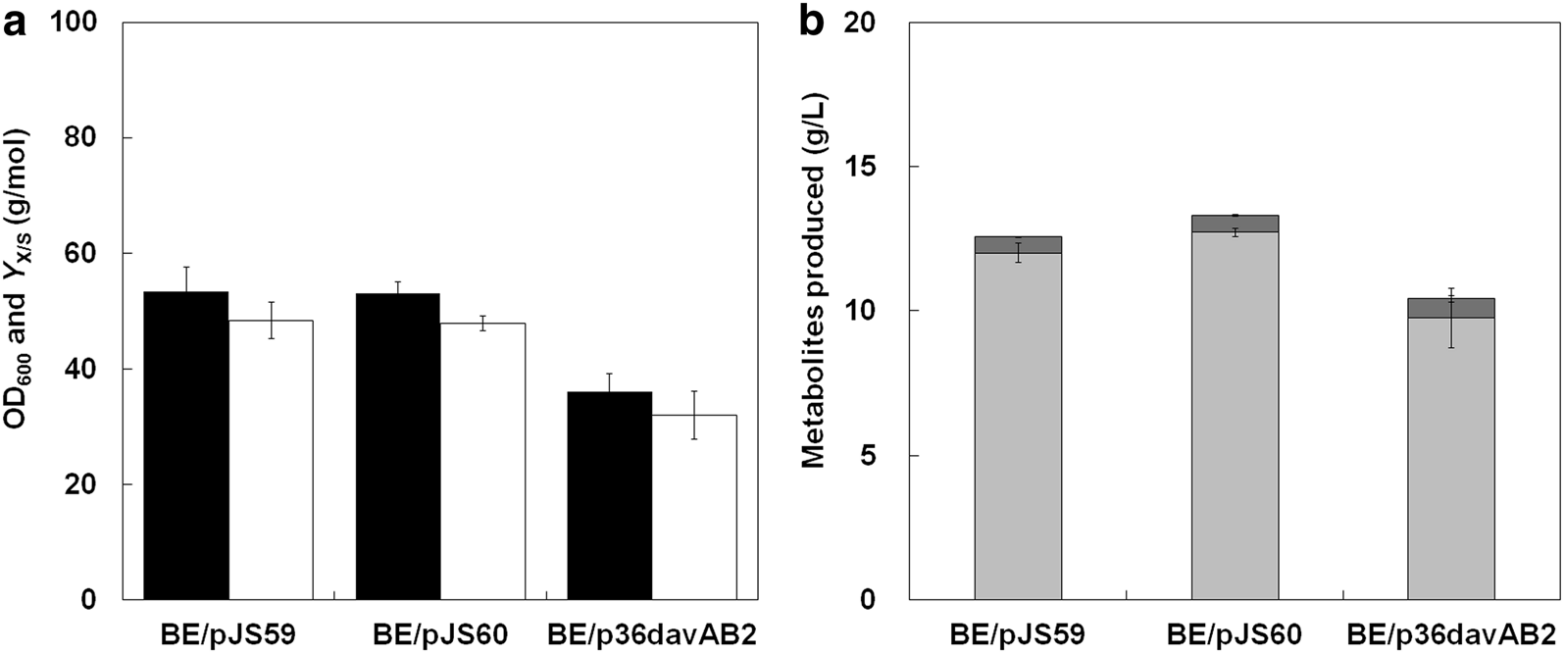
Growth and production characteristics of the *C*. *glutamicum* BE strain harboring the pJS59, pJS60, or p36davAB2 vector after 44 h of shake-flask cultivation (*n* = 3, *error bars* = SD). The *C*. *glutamicum* BE strain containing no plasmids was used as a non-engineered control. **a** The final OD_600_ at the end of cultivation is shown for all strains tested, and an experimentally determined correlation factor (0.28) was used to determine the biomass yield (*Y*_X/S_). **b** The production characteristics include the final titers for l-lysine (*light-grey bars*), 5AVA (*dark-grey bars*), and glutaric acid (*black bars*)

In addition to the *tac* promoter used in pEKEx1, promoters for SOD (NCgl2826; E.C. 1.15.1.1) and the transcription factor Tu (Tuf; Ncgl0480; E.C. 3.6.5.3) have also been widely used in metabolic engineering of *C*. *glutamicum* because of their capabilities to support strong gene expression at the chromosome level [34]. Although the *sod* promoter is known to exhibit weaker plasmid-based expression than the *tac* promoter [35], weak and medium-strength expression driven by constitutive promoters might be more effective than higher expression levels for producing chemicals of interest under different circumstances [15]. Moreover, use of the constitutive promoters circumvents the requirement for costly additives such as IPTG. Therefore, we replaced the *lacI* ^*Q*^ gene and *tac* promoter in pJS38 with P_sod_ or P_tuf_ (Additional file 5: Table S1). These constructs were then introduced into the *C*. *glutamicum* BE strain, and their functions were investigated by flask cultivation. However, expression of the *davAB* genes under control of the *sod* promoter (pJS59) and *tuf* promoter (pJS60) did not result in higher 5AVA production relative to that produced by the parent construct, pJS38 containing the *tac* promoter (Figs. 2, 4). *C*. *glutamicum* BE (pJS59) and *C*. *glutamicum* BE (pJS60) produced 556 and 587 mg/L 5AVA, respectively, with no glutaric acid observed in the culture media after 44 h of flask cultivation.

We also investigated the newly designed synthetic promoter active in *C. glutamicum*, the H36 promoter [36], to see if it can possibly improve 5AVA production; it was successfully employed for the expression of glutamate decarboxylase (GAD) and lysine decarboxylase (LDC) in *C. glutamicum* strains, resulting in high-level production of gamma-aminobutyrate (GABA) and cadaverine, respectively [30, 37].

We replaced the *lacI* ^*Q*^ and the *tac* promoter in pJS38 with the P_H36_ promoter to construct p36davAB2. However, cultivation of *C. glutamicum* BE harboring p36davAB2 also did not improve 5AVA production, which resulted in production of 661 mg/L 5AVA under the same culture condition (Fig. 4). These results indicate that expression of the *davAB* genes was still not strong enough to enable efficient conversion of l-lysine to 5AVA.

**Fig. 5:**
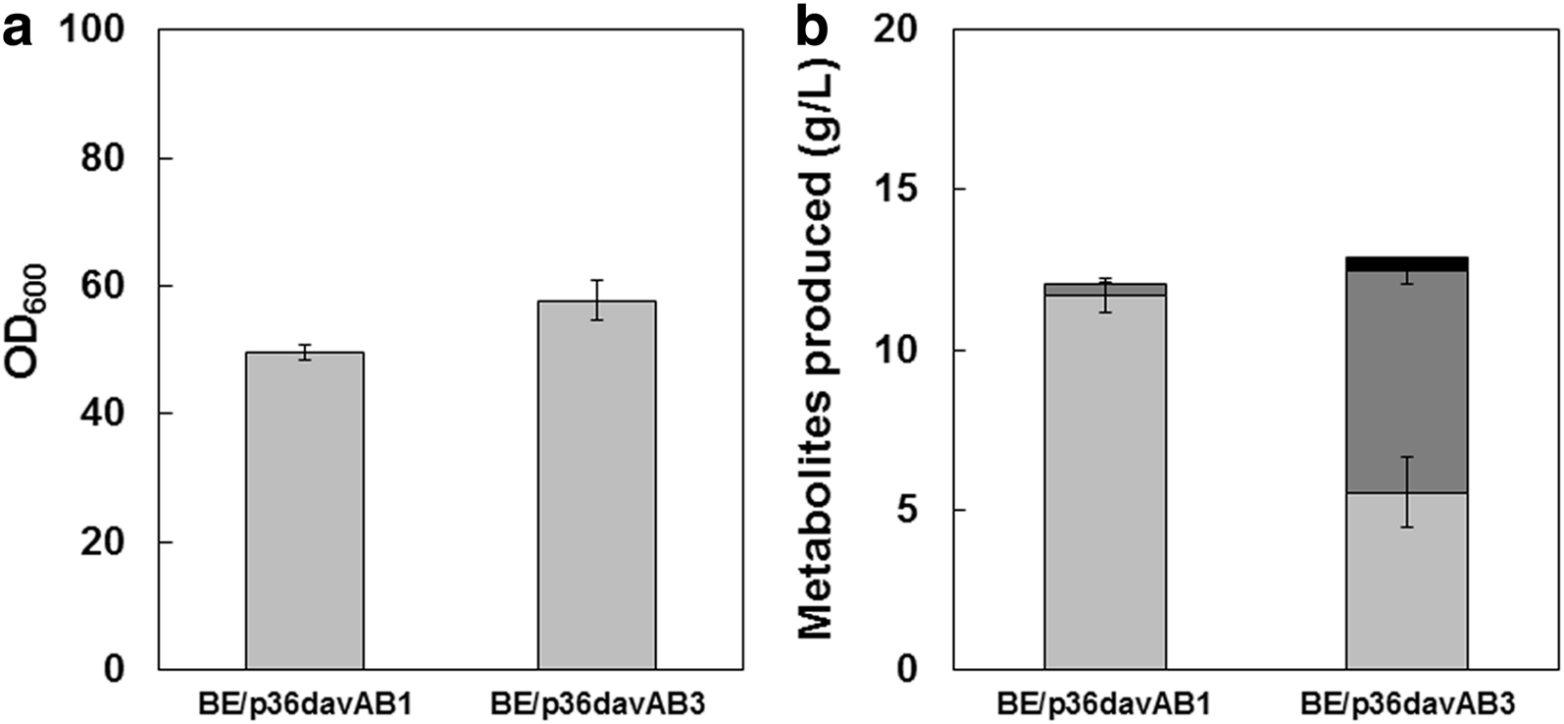
Growth and production characteristics of the *C*. *glutamicum* BE strain harboring p36davAB1 or p36davAB3 after 44 h of shake-flask cultivation (*n* = 3, *error bars* = SD). **a** The final OD_600_ at the end of cultivation is shown for the strains tested. **b** The production characteristics include the final titers for l-lysine (*light-grey bars*), 5AVA (*dark-grey bars*), and glutaric acid (*black bars*)

To continue exploring expression vector systems for possible improvement of 5AVA titer, we noticed that the strong H36 promoter originally developed with a different backbone vector, pCES208 [36, 38], might not be optimal for pEKEx1. Engineered *C. glutamicum* strains harboring a pCES208-based plasmid for expression of target genes under strong synthetic promoters, such as H30 and H36, have been reported to efficiently produce GABA and cadaverine from renewable resources [30, 37]. Therefore, we transferred codon-optimized versions of the *davAB* genes into the pCES208 vector system. The new construct, p36davAB1, was further modified by inserting a His_6_-Tag into the N-terminal of *davA* gene, resulting in p36davAB3. This was done because there have been reports showing that His_6_-tagged constructs can sometimes be expressed more efficiently [39, 40]. These constructs were transformed into the *C*. *glutamicum* BE strain and assessed by flask cultivation. Whereas *C*. *glutamicum* BE (p36davAB1) produced only 0.4 g/L 5AVA along with 11.7 g/L l-lysine, *C*. *glutamicum* BE (p36davAB3) produced 6.9 g/L 5AVA, with 5.5 g/L l-lysine remaining unconverted (Fig. 5). The 5AVA concentration obtained represents a 130 % increase over that (Fig. 2) obtained with *C*. *glutamicum* BE (pJS38). Interestingly, the construct containing the His_6_-tagged variant produced substantially more 5AVA compared to that produced using the construct lacking the His-tag, possibly because of the improved stability afforded by the 5′ modification, which resulted in higher expression of the *davAB* genes in the recombinant *C. glutamicum* BE strain (Additional file 1: Figure S1). Comparison of mRNA folding energies (ΔG) with the RNA secondary structure prediction program Mfold (http://unafold.rna.albany.edu/?q=mfold/download-mfold) suggested that the ΔG for the first 30 nucleotides starting from the +1 site of the H36 promoter in p36davAB2 is −6.00 kcal/mol, which is much lower than ΔG of −0.06 kcal/mol obtained in p36davAB3. The higher ΔG in p36davAB3 indicates that less stable mRNA produced by p36davAB3 might allow the translation machinery to bind more easily than much stable mRNA produced by p36davAB2.

### Examination of 5AVA production by engineered *C*. *glutamicum* expressing the *davAB* genes under the control of the strong H36 promoter in fed-batch fermentation

Having achieved improved 5AVA production in flask culture, fed-batch culture of *C*. *glutamicum* BE (p36davAB3) was performed next in a 5-L fermentor. *C*. *glutamicum* BE (p36davAB3) produced 19.7 g/L 5AVA in 157 h, with the overall yield and productivity of 0.08 g/g and 0.16 g/L/h, respectively (Fig. 3d). This strain also accumulated 13.4 g/L glutaric acid as a byproduct at the end of the cultivation. On the other hand, l-lysine accumulation decreased significantly compared to that observed with *C*. *glutamicum* BE (pJS38). l-Lysine accumulated to 3.7 g/L in 25 h, but production remained between 1 and 2 g/L over the entire cultivation period (Fig. 3d). Citric acid was another major byproduct, but its concentration remained at ~1 g/L throughout cultivation. Notably, the production patterns observed during fed-batch fermentation were different from those observed during flask cultivation. Although large portions of l-lysine remained unconverted at the end of the flask cultivation, very little l-lysine remained in fed-batch fermentation. This indicates that control of pH and provision of sufficient air streams were beneficial for 5AVA production and provided better results during fed-batch fermentation. A sufficient air supply is important for cultivation because lack of sufficient air can result in accumulation of substantial concentrations of lactic and acetic acids in the fermentation broth [30, 41]. These byproducts were not observed in our cultivation conditions, contrary to previous reports. These results suggest that the strategy combining improved expression of the *davA* gene fused with His_6_-Tag at its N-Terminal and the *davB* gene as an operon under control of the strong synthetic H_36_ promoter was successful in directing most of the l-lysine pool toward 5AVA, resulting in efficient production of 5AVA.

### Construction of an engineered *C. glutamicum gabT* mutant for enhanced production of 5AVA with greatly reduced glutaric acid production

While 19.7 g/L of 5AVA could be produced by fed-batch cultivation of *C*. *glutamicum* BE (p36davAB3), glutaric acid, a major byproduct, was still produced to a relatively high concentration (up to 13.4 g/L). In order to further enhance 5AVA production, conversion of 5AVA into glutaric acid should be minimized. However, no enzyme responsible for converting 5AVA into glutaric acid is known in *C. glutamicum*. Thus, we performed molecular-docking simulations, which suggested possible interactions between endogenous GabT and 5AVA (Additional file 2: Figure S2, Additional file 3: Figure S3). GabT shares homology (60 % by primary peptide structure) with 4-aminobutyrate aminotransferase (SGR_1829) in *Streptomyces griseus*, which exhibits 60 % relative aminotransfer activity for 5AVA [42]. Although the pyridoxal phosphate moiety was in the correct orientation and position, the orientation of bound 5AVA was twisted, possibly because of the larger size of the substrate being accommodated in the active site. Additionally, the enzyme also shared high homology (Additional file 3: Figure S3) with *P*. *putida* DavT, which binds 5AVA as its natural substrate (Additional file 4: Figure S4). The major difference in active sites between GabT and DavT is that DavT contains a glutamine residue (Gln80) rather than a methionine residue, enabling accommodation of the *ω*-amino group in the binding pocket (Additional file 3: Figure S3). However, GabT from *S*. *griseus*, even with the methionine residue at this position, is sufficiently promiscuous to accept 5AVA as a substrate [42]. Thus, we could conclude from the docking simulations that the endogenous *C. glutamicum* GabT might accommodate 5AVA as a substrate for aminotransfer reactions, leading to the formation of glutaric acid.

**Fig. 6:**
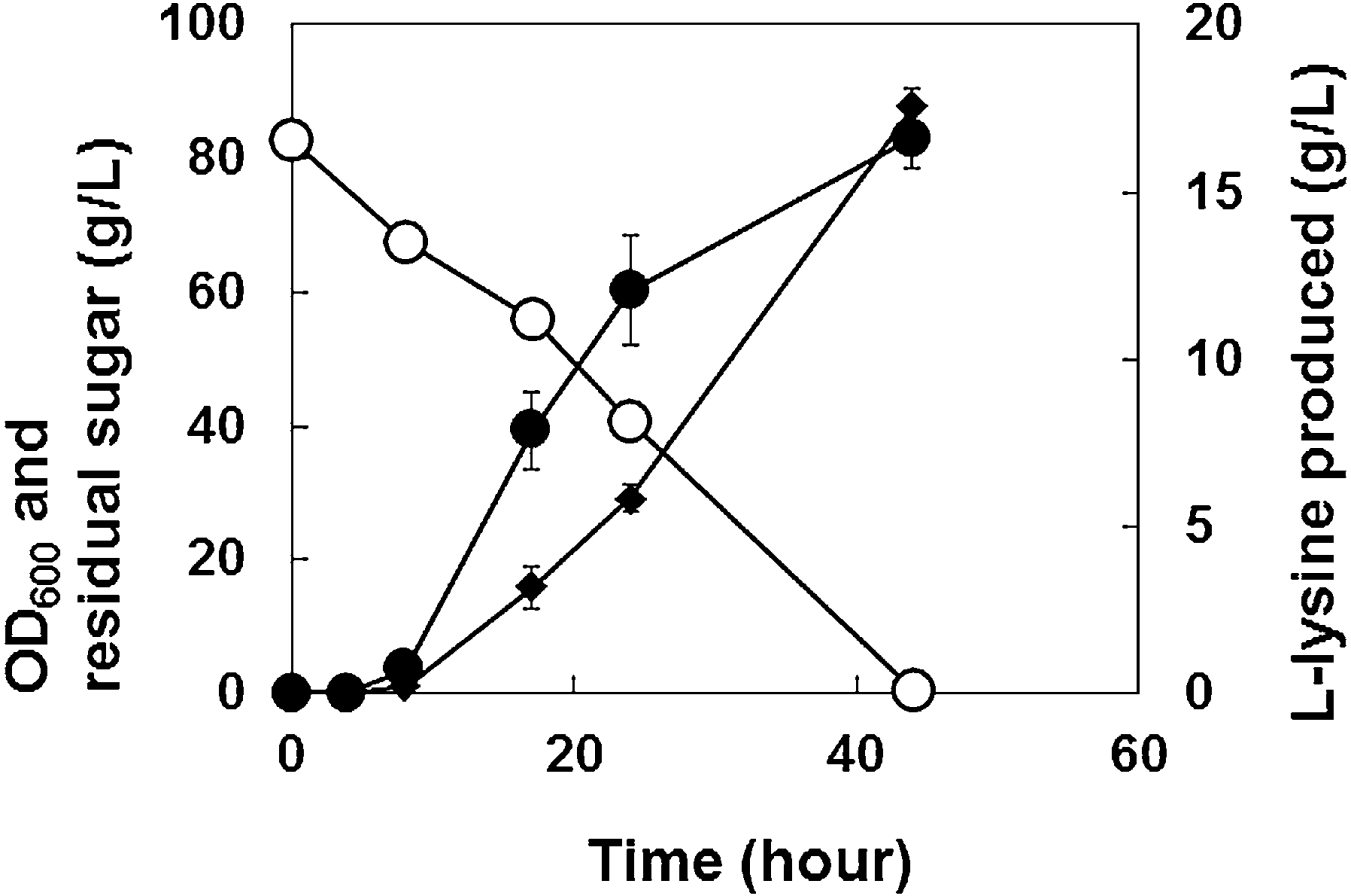
Growth and production characteristics of the AVA2 strain for 44 h of shake-flask cultivation (*n* = 3, *error bars* = SD). OD_600_ (*filled circle*), glucose (*empty circle*) and l-lysine (*filled diamond*) are shown

**Fig. 7:**
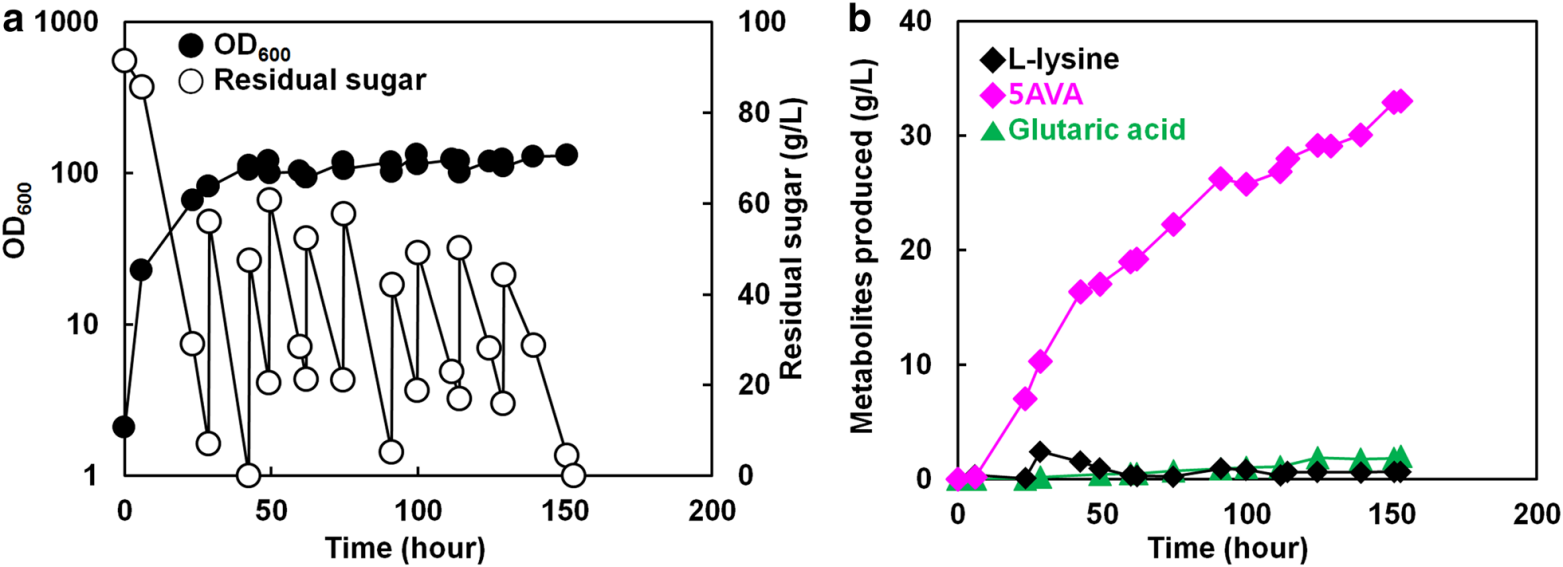
Fed-batch cultivation of *C*. *glutamicum* AVA2 harboring p36davAB3 for the production of 5AVA in laboratory-scale bioreactor from glucose. **a** Characteristics of the fed-batch cultivation profile including growth (*filled circles*, OD_600_), residual sugar (*empty circles*; g/L) and **b** production titers of products including l-lysine (*dark diamonds*), 5AVA (*magenta diamonds*), and glutaric acid (*green triangles*) are plotted against the cultivation time

Based on the above results, the *gabT* gene (E.C. 2.6.1.19, encoding 4-aminobutyrate aminotransferase, Ncgl0462) was deleted from the chromosome of *C. glutamicum* BE to construct *C*. *glutamicum* AVA2. *C*. *glutamicum* AVA2 produced 17.5 g/L of l-lysine by flask cultivation in 44 h, with no residual 5AVA detected (Fig. 6). This result suggests that deletion of the *gabT* gene did not inhibit cell growth and l-lysine production. Plasmid p36davAB3 was then transformed into *C*. *glutamicum* AVA2 to assess 5AVA production. Fed-batch cultivation of engineered *C*. *glutamicum* AVA2 (p36davAB3) in a 5-L fermenter resulted in production of 33.1 g/L 5AVA with greatly reduced glutaric acid (2.0 g/L) and l-lysine (648.3 mg/L) at the end of cultivation. The overall yield and productivity obtained were 0.1 g/g glucose (163.1 mmol/mol) and 0.22 g/L/h, respectively (Fig. 7). Cells grew to an OD_600_ of 134 in 153 h, with a measured dry cell weight of 36.1 g/L, with the maximum specific growth rate was 0.4 h^−1^. The maximum specific 5AVA productivity was 65.8 mg/g/h, which gradually decreased to 22.1 mg/g/h at the end of cultivation. Additionally, the l-lysine concentration peaked at 28 h, but remained as low as 1 g/L for the remainder of the cultivation. As expected, *gabT* deletion resulted in a significant decrease in glutaric acid production compared to that observed in the parent strain. However, the continued presence of glutaric acid in the culture broth suggests that unknown aminotransferases still remain in *C*. *glutamicum* that are capable of converting 5AVA to glutaric acid, although at lower efficiencies than GabT.

## Conclusions

In this study, we report development of engineered *C. glutamicum* strains for the production of 5AVA from glucose. Expression of two key enzymes, lysine 2-monooxygenase and delta-aminovaleramidase, was systematically optimized by examining different promoters, origins of replication, codon usage of the *davAB* genes, and even 5′ modification of the *davA* gene with a His-tag, all of which were found to be important for determining the optimal and stable plasmid-based expression of the *davAB* genes in *C. glutamicum*. In addition, production of a major byproduct, glutaric acid, could be significantly reduced by identifying previously unknown enzyme GabT responsible for converting 5AVA to glutaric acid and deleting the corresponding gene from the chromosome. Fed-batch cultivation of the final engineered *C*. *glutamicum* AVA2 strain harboring p36davAB3 produced 33.1 g/L 5AVA with greatly reduced glutaric acid (2.0 g/L). The metabolically engineered *C. glutamicum* strains developed in this study should be useful for enhanced fermentative production of the novel C5 platform chemical, 5AVA, from renewable resources such as glucose.

## Methods

### Strains and plasmids

All bacterial strains and plasmids used in this study are listed in Additional file 5: Table S1. All DNA manipulations were performed following standard procedures [43]. Primers used in this study (Additional file 6: Table S2) were synthesized at Bioneer (Daejeon, Korea). *C. glutamicum* BE (KCTC 12390BP) was used as the base strain for 5AVA production. Polymerase chain reaction (PCR) was performed with the C1000 Thermal Cycler (Bio-Rad, Hercules, CA, USA). The general PCR condition for amplifications of target genes using primer sets listed in Additional file 6: Table S2 is as follows: 1 cycle of 95 °C for 5 min; 30 cycles of 94 °C for 30 s, 52 °C for 30 s, 72 °C for 1 min 30 s; and a final extension of 72 °C for 5 min. The final reaction volume is 20 μL. The in-house-developed *C*. *glutamicum* shuttle vector pKCA212-MCS was constructed by cloning the origin of replication of the cryptic plasmid pCC1 [33] into pKA212-MCS at the *Aat*II and *Xho*I sites. The origin of replication of pCC1 was synthesized by GenScript (http://www.genscript.com) based on the reported sequence. Plasmid pKA212-MCS was constructed by replacing the chloramphenicol-resistance gene of pKA312-MCS [11] with a kanamycin-resistance gene obtained from pZA21-MCS (http://www.expressys.com) by restriction digest with *Aat*II and *Spe*I. The *davAB* genes from pKE112-DavAB [20, 21] were restriction-digested and ligated into pKCA212-MCS to construct pKCA212davAB using the same restriction enzyme sites (*Eco*RI/*Kpn*I, *Kpn*I/*Bam*HI). A 16-bp untranslated region (tttcacacaggaaaca) containing a ribosome-binding site was present between the two genes for *davB* expression. The same genes were also cloned into pEKEx1 to construct pJS30. The codon-optimized versions of *davAB* genes (Additional file 7: Table S3) with preferred codon usage in *C*. *glutamicum* were synthesized by Bioneer (Daejeon, Korea) and cloned into the *Eco*RI/*Bam*HI restriction enzyme sites in pEKEx1 to yield pJS38.

To construct promoter variants of pEKEx1, promoterless pEKEx1 was created by removing the *tac* promoter and the initial 778 bp of the coding sequence of the *LacI* ^*Q*^ gene from pEKEx1 by restriction digestion with *Eco*RV/*Eco*RI. The desired promoters were similarly designed as previously described [5, 34, 44] and inserted into the promoterless pEKEx1 vector. The region 250 bp upstream of the start codon for the superoxide dismutase gene (NCgl2826, E.C. 1.15.1.1) was amplified by polymerase chain reaction (PCR) from *C*. *glutamicum* ATCC 13032 chromosome using primers Psod_F_EcoRV and Psod_R_EcoRI, and then digested and cloned into the *Eco*RV/*Ec*oRI sites of the promoterless pEKEx1 vector to construct pJS57. The 248-bp sequence upstream of the start codon for the gene encoding the elongation factor Tu (Ncgl0480, E.C. 3.6.5.3) was amplified by PCR from *C*. *glutamicum* ATCC 13032 chromosome using the primers Ptuf_F_EcoRV (v2) and Ptuf_R_EcoRI, and then digested and cloned into the promoterless pEKEx1 vector to yield pJS58. The codon-optimized *davAB* genes from pJS38 were restriction-digested with *Eco*RI/*Bam*HI and cloned into the pJS57 and pJS58 vectors at the *Eco*RI/*Bam*HI restriction enzyme sites to make pJS59 and pJS60, respectively.

Plasmid p36davAB2 was constructed from pEKEx1 by cloning the codon-optimized *davAB* genes. Promoterless pEKEx1 was constructed by methods similar to those described in the previous paragraph, except that the genes were cloned into the *Eco*RV/*Pst*I restriction sites of the vector. The P_H36_ promoter was amplified by PCR using the JW02H-F and JW02H-R primers from pCES208H36GFP, and the fragments were restriction-digested with *Eco*RV/*Eco*RI. A second round of PCR using primers JW02AB-F and JW02AB-R from pJS38 generated codon-optimized *davAB* gene fragments that were restriction-digested with *Eco*RI/*Pst*I. The resulting products were then ligated into the *Eco*RV/*Pst*I restriction sites of the promoterless pEKEx1 vector to yield p36davAB2.

Plasmid p36davAB1 was constructed from pCES208 by cloning the codon-optimized *davAB* genes. Products from the first round of PCR using primers JW01A-F and JW01A-R were used for amplification of the *davA* gene, which was then restriction-digested with *Bam*HI/*Sfi*I. The second round of PCR used primers JW01B-F and JW01B-R to amplify the *davB* gene, which was then restriction-digested with *Not*I. These fragments were cloned into the pCES208H36GFP vector [36] by replacing the *egfp* gene to yield p36davAB1.

Plasmid p36davAB3 was constructed from the pCES208H36EGFP vector [36]. The codon-optimized *davA* gene fused with His_6_-Tag at its N-Terminal was amplified using primers JW03A-F and JW01A-R and restriction-digested with *Bam*HI and *Sfi*I. The codon-optimized *davB* gene was amplified using primers JW01B-F and JW03B-R and restriction-digested with *Not*I. The two products were then cloned into the pCES208H36EGFP vector by replacing the *egfp* gene to construct p36davAB3.

Plasmid pJS113 *beta* was constructed from the pK19mobsacB vector [45]. Primers 113 i1F *beta* and 113 i1R *beta* were used to PCR-amplify the upstream region and a portion of the *gabT* gene from *C*. *glutamicum.* Primers 113 i2F *beta* and 113 i2R *beta* were then used to PCR-amplify the downstream region and a portion of the *gabT* gene of *C*. *glutamicum.* The two PCR products were joined by a third PCR using primers 113 i1F *beta* and 113 i2R *beta*. The final PCR product was cloned into the *Pst*I-digested pK19mobsacB to make pJS113 *beta*. pJS113 *beta* was subsequently used to disrupt the *gabT* gene in the *C*. *glutamicum* BE chromosome, resulting in the strain *C*. *glutamicum* AVA2. This in-frame deletion left a 330-bp deletion in the 280–609 region of the 1347-bp *gabT* gene.

*E*. *coli* DH5*α* and TOP10 strains (Additional file 5: Table S1) were used for general cloning purposes. All constructed plasmids introduced into *C*. *glutamicum*, except for pJS113 *beta*, were prepared in unmethylated form using the methylation-deficient *E*. *coli* JM110 strain (Stratagene; Agilent Technologies, Santa Clara, CA, USA). pJS113 *beta* was propagated in *C*. *glutamicum* by bacterial conjugation using *E*. *coli* S17-1 as a donor [45]. Plasmids were introduced via electroporation as previously described [46]. Cells were transferred to a microcuvette and electroporated using a micropulser. Cells were transformed with about 2 μg of DNA by electroporation (1.8 V and 400 Ω). Pre-chilled preculture medium (900 μL) was added and the transformed cells were allowed for growth recovery for 2 h without shaking in a 30 °C incubator. The transformed cells were then spread onto the agar plates containing kanamycin as a selective marker.

### Culture media

Cells were cultured in media described below, the compositions of which were modified from previous reports [34, 43]. The pre-culture medium for shake-flask cultivation consisted of 10 g/L beef extract (BD Bacto, Franklin Lakes, NJ, USA), 40 g/L brain–heart infusion (BD Bacto), 20 g/L d-sorbitol, and 10 g/L glucose [41]. The flask culture medium (pH 7.2) consisted of 80 g/L glucose, 1 g/L MgSO_4_, 1 g/L K_2_HPO_4_, 1 g/L KH_2_PO_4_, 1 g/L urea, 20 g/L (NH_4_)_2_SO_4_, 10 g/L yeast extract, 100 μg/L biotin, 10 mg/L β-alanine, 10 mg/L thiamine HCl, 10 mg/L nicotinic acid, 1.3 mg/L (NH_4_)_6_MoO_24_, 40 mg/L CaCl_2_, 10 mg/L FeSO_4_, 10 mg/L MnSO_4_, 5 mg/L CuSO_4_, 10 mg/L ZnSO_4_, and 5 mg/L NiCl_2_.

For fermentation experiments, the seed medium (pH 7.0) consisted of 20 g/L glucose, 1 g/L MgSO_4_, 10 g/L beef extract, 1 g/L K_2_HPO_4_, 1 g/L KH_2_PO_4_, 0.5 g/L urea, 10 g/L yeast extract, 100 μg/L biotin, 200 μg/L thiamine HCl, 10 mg/L FeSO_4_, and 10 mg/L MnSO_4_. The fermentation medium (1.8 L) contained per liter: 160 g of glucose, 2 g of MgSO_4_, 2 g of K_2_HPO_4_, 2 g of KH_2_PO_4_, 2 g of urea, 40 g of (NH_4_)_2_SO_4_, 20 g of yeast extract, 50 mg of CaCl_2_, 50 μg of biotin, 20 mg of β-alanine, 20 mg of thiamine HCl, 20 mg of nicotinic acid, 1.3 mg of (NH_4_)_6_Mo_7_O_24_, 10 mg of FeSO_4_, 10 mg of MnSO_4_, 5 mg of CuSO_4_, 10 mg of ZnSO_4_, 5 mg of NiCl_2_, and 1 mL of antifoam reagent (Antifoam 204; Sigma-Aldrich, St. Louis, MO, USA). Each feeding solution (200 mL) contained 90 g of glucose.

### Flask cultivation

Stock cells stored in glycerol were used to inoculate 5-mL pre-cultures, which were grown at 30 °C with shaking at 200 rpm in an incubator (JSSI-300C; JS Research Inc., Gongju, Korea) for 17–18 h. Cells suspended in 250-μL aliquots of pre-culture were harvested by centrifugation (Centrifuge 5415 D; Eppendorf, Hamburg, Germany) and transferred to a 25-mL primary culture in autoclaved 300-mL baffled Erlenmeyer flasks, each containing 1.5 g of CaCO_3_ to maintain the pH at ~7.0 during cultivation. Primary cultures were grown with shaking in an incubator for 44 h. When appropriate, isopropyl-β-d-thiogalactopyranoside (IPTG) at a final concentration of 0.5 mM was used to induce gene expression during the early log phase (OD_600_ = 0.5–0.6), with 25.0 μg/mL kanamycin added for selective pressure (Ravasi et al. [35]).

### Fed-batch fermentation

Stock cells stored in glycerol were used to inoculate 5.0-mL pre-cultures, which were grown at 30 °C with shaking in an incubator for 17–18 h. Two 1-mL samples of the pre-culture were transferred to two 1-L Erlenmeyer flasks, each containing 100 mL of seed medium, and grown with shaking (200 rpm) in a 30 °C incubator for 19–20 h. The entire seed culture (200 mL) was added as the inoculum to the 1.8-L primary culture in a fermenter (initial OD_600_ = 1.5–2.0 in 2 L). IPTG at a final concentration of 1 mM and kanamycin (25.0 μg/L) were also added during fermentation inoculation. A NBS BioFlo 3000 fermenter system (New Brunswick Scientific, Edison, NJ, USA) equipped with a 6.6-L jar was used for all fed-batch cultivation experiments. The pH was maintained at 7.0 by addition of 28 % (v/v) ammonia solution (Junsei Chemical Co., Ltd., Tokyo, Japan). Temperature and agitation were maintained at 30 °C and 600 rpm, respectively, by a proportional-integral-derivative controller throughout the entire cultivation period. The aeration rate was maintained at 1 L/L/min. Foaming was suppressed by addition of 1:10 diluted antifoam 204 (Sigma-Aldrich). Feeding solution (200 mL) was manually added each time the residual glucose level decreased to <20 g/L.

### Analytical procedures

Two high-performance liquid chromatography (HPLC) systems, Agilent 1100 (Agilent Technologies) and Waters Breeze 2 (Waters Corporation, Milford, MA, USA), were used to determine the metabolite concentration in the culture broth. For detection of amino compounds, the supernatant of the culture samples was reacted with *o*-phthaldehyde as previously described [13] prior to injection into the Eclipse Zorbax-AAA column (Agilent Technologies). Linear gradients of mobile phase A [10 mM Na_2_HPO_4_, 10 mM Na_2_B_4_O_7_·10H_2_O, and 8 mg/L NaN_3_ (pH 7.2)] and mobile phase B (methanol, acetonitrile, and water at a volumetric ratio of 45:45:10) were used to separate the amino acids in the column. Borate buffer (0.4 M; pH 10.2) was used as a buffering agent rather than pH 9.0 buffer as previously described [13]. The derivatized compounds were detected using a diode-array detector at 338 nm. The column temperature was set to 25 °C, and the flow rate of the pump was set to 0.640 mL/min. The following gradient was applied for resolving the compounds: 0–0.5 min, 0 % B; 0.5–18 min, a linear gradient of B from 0 to 57 %; 18–26 min, a linear gradient of B from 57 to 100 %; 26–31.8 min, 100 % B; 31.8–31.9 min, a linear gradient of B from 100 to 0 %; 31.9–32 min, 0 % by volume. Glutaric acid was detected using the Waters Breeze 2 HPLC system (Waters Corporation) with a MetaCarb 87H column (Varian; Crawford Scientific, Strathaven, UK) and a constant flow of sulfuric acid solution at 0.5 mL/min. The Waters Breeze 2 system included an isocratic pump (Waters 1515; Waters Corporation), a refractive index detector (Waters 2414; Waters Corporation), and an autosampler (Waters 2707; Waters Corporation).

Cell growth was monitored by measuring the OD_600_ with an Ultrospec 3000 spectrophotometer (Amersham Biosciences, Uppsala, Sweden). The correlation factor (0.28 g of dry weight of cells per L per OD_600_ of 1) was experimentally determined and used for biomass concentration calculation of flask-cultivated cells. This correlation factor was in agreement with a previously reported value [47]. Glucose concentration was measured using a 2700 biochemistry analyzer (YSI, Yellow Springs, OH, USA). When necessary, diluted HCl solution was used to neutralize CaCO_3_ in the cultivation media.

### Molecular docking simulation

Molecular docking simulations were performed using Autogrid and Autodock 4.2.5.1 software [48]. Gasteiger charges and hydrogen atoms were added using AutoDockTools 1.5.6. A Lamarckian genetic algorithm with default parameters was used, and no peptide residues were kept flexible. The docking grid was set to encompass the catalytic pocket, but not the entire enzyme. For docking of the natural substrate of 4-aminobutyrate aminotransferase, the substrate molecules were separately saved from a known structure (PDB ID: 4ATQ) [49] as a single molecule in the form of an external aldimine and used as a ligand. For docking with 5AVA aminotransferase, pyridoxal phosphate and 5AVA in the form of an external aldimine were used as the ligand. Torsion about the bond between the pyridine moiety of the pyridoxal phosphate and the Schiff base was not allowed during docking. The docking results were visualized using PyMol 1.6 (https://www.pymol.org/pymol) without additional hydrogen atoms.

### Molecular modeling

Homology modeling was carried out with SWISS-MODEL [50]. 4-Aminobutyrate aminotransferase (E.C. 2.6.1.19) of *C. glutamicum* was homology modeled using the same enzyme from *A. aurescens* (PDB ID: 4ATQ chain B) [49] as a template. A minor correction was applied for this model (Additional file 2: Figure S2) based on a different enzyme structure (PDB ID: 3LV2). The same enzyme from *S. griseus* was homology modeled using that from *Mycobacterium smegmatis* (PDB ID: 3Q8 N chain D) as a template. 5-Aminovalerate aminotransferase (E.C. 2.6.1.48) of *P. putida* KT2440 was homology modeled using 4-aminobutyrate aminotransferase from *E. coli* (PDB ID: 1SFF chain A) [51] as a template.

## Additional files

10.1186/s12934-016-0566-8 Expression levels of the *davA* and *davB* genes in recombinant *C. glutamicum* BE harboring p36davAB1, p36davAB2, and p36davAB3, all of which were examined by electrophoresis on a 12 % (w/v) sodium dodecylsultate-polyacrylamide gel (SDS-PAGE). T and S stand for total fraction and soluble fraction, respectively.

10.1186/s12934-016-0566-8 Molecular docking with *C*. *glutamicum* GabT. Selected residues in the PLP-binding pocket from the SWISS-MODEL-predicted molecular structure of *C*. *glutamicum* GabT (magenta) are shown. The Ile261 residue (orange) near the predicted binding pocket for PLP from the original modeling simulation result protrudes into the binding pocket, thereby hindering subsequent docking simulations. This residue was corrected based on a known crystal structure of a different enzyme (yellow; PDB ID: 3LV2) with the correct Ile orientation at the same position.

10.1186/s12934-016-0566-8 Molecular docking of external aldimine with *C*. *glutamicum* GabT. (A) Molecular docking of external aldimine (grey; PLP and *γ*-aminobutyrate) to homology-modeled GabT from *C*. *glutamicum* (magenta). A known crystal structure of GabT from *Arthrobacter aurescens* (green, PDB ID: 4ATQ) (Bruce et al. [49]) is superimposed for comparison. (B) Molecular docking of external aldimine (blue; PLP and AVA) to homology-modeled GabT from *C*. *glutamicum* (magenta). Homology-modeled DavT from *P*. *putida* (salmon) and homology-modeled GabT from *S*. *griseus* (cyan) are superimposed for comparison.

10.1186/s12934-016-0566-8 Simulation of molecular docking of 5AVA to homology-modeled DavT from *P*. *putida* KT2440. DavT was homology-modeled based on a known structure (PDB ID: 1SFF).

10.1186/s12934-016-0566-8 Strains and plasmids used in this study.

10.1186/s12934-016-0566-8 Primers used in this study.

10.1186/s12934-016-0566-8 Sequence of codon optimized *davA* and *davB* genes from *P. putida.*

## Abbreviations

5AVA: 5-aminovalerate
SOD: superoxide dismutase
IPTG: isopropyl-β-d-thiogalactopyranoside
