# Supplementary figures and images for: Metabolic engineering of Corynebacterium glutamicum for enhanced production of 5-aminovaleric acid
Source: Microb Cell Fact. 2016 Oct 7;15:174. doi: 10.1186/s12934-016-0566-8 (PMC5054628; doi:10.1186/s12934-016-0566-8)

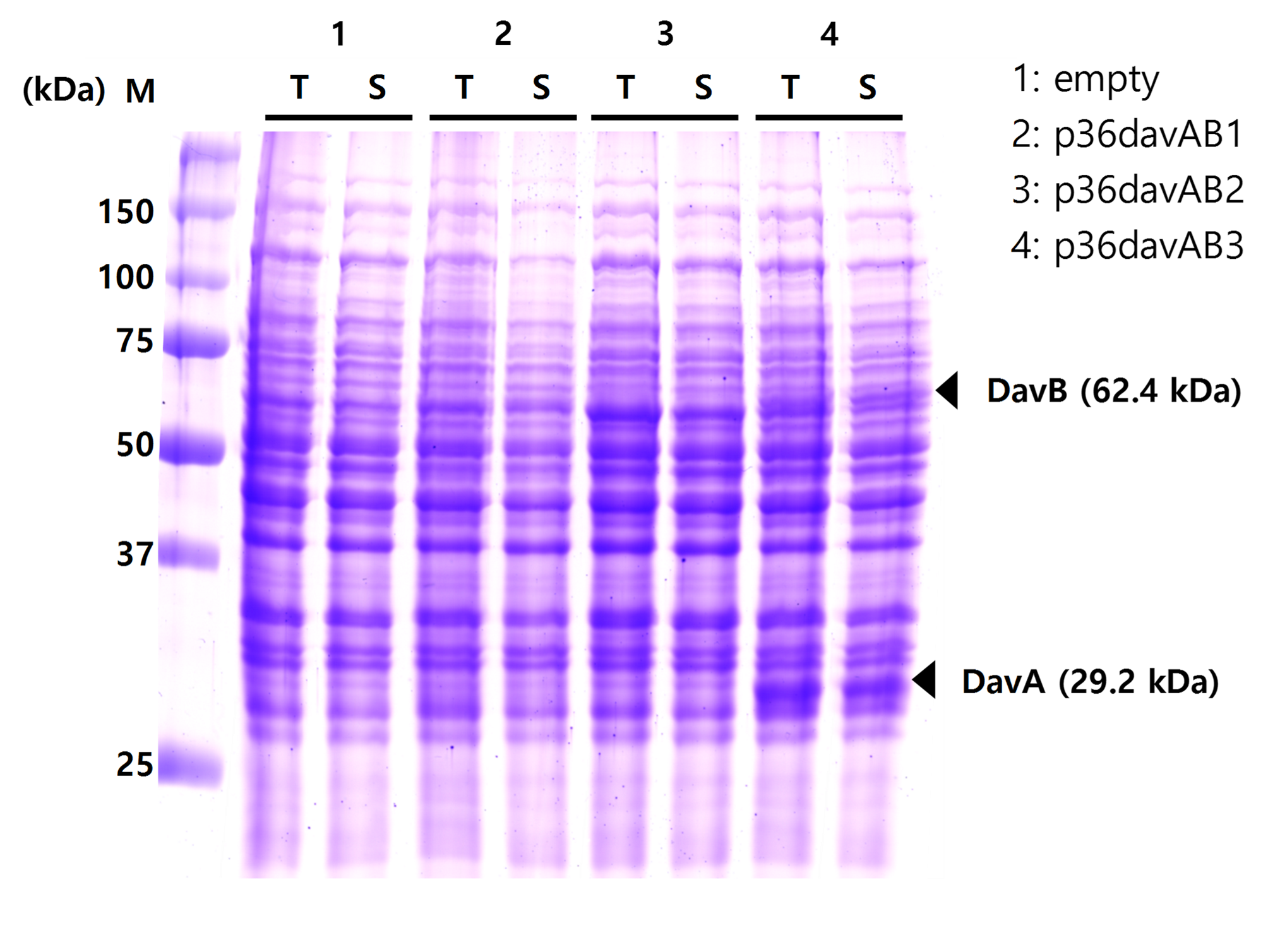

Supplement: Supplementary file 1 — 10.1186/s12934-016-0566-8 Expression levels of the davA and davB genes in recombinant C. glutamicum BE harboring p36davAB1, p36davAB2, and p36davAB3, all of which were examined by electrophoresis on a 12 % (w/v) sodium dodecylsultate-polyacrylamide gel (SDS-PAGE). T and S stand for total fraction and soluble fraction, respectively. [file 12934_2016_566_MOESM1_ESM.tif]

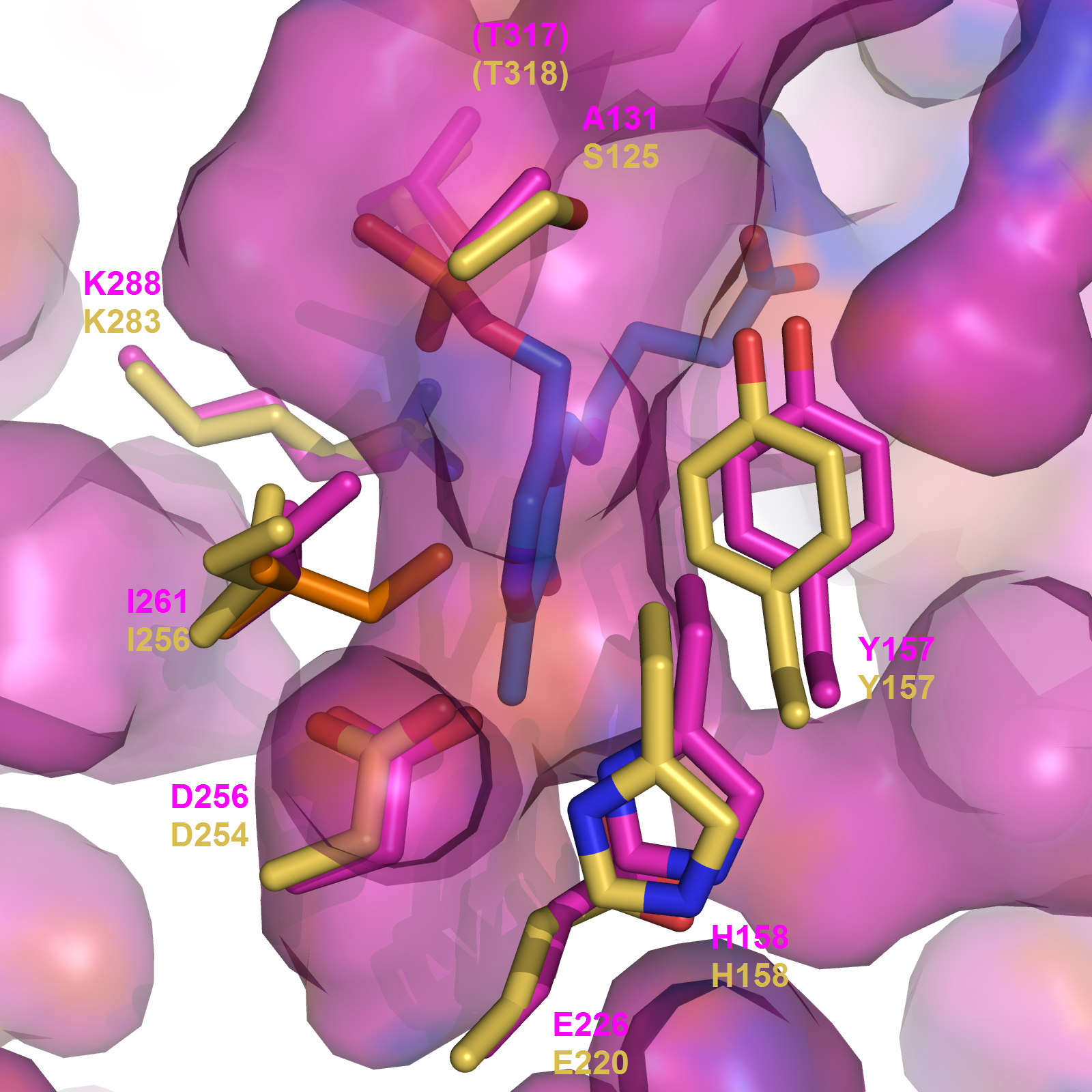

Supplement: Supplementary file 2 — 10.1186/s12934-016-0566-8 Molecular docking with C. glutamicum GabT. Selected residues in the PLP-binding pocket from the SWISS-MODEL-predicted molecular structure of C. glutamicum GabT (magenta) are shown. The Ile261 residue (orange) near the predicted binding pocket for PLP from the original modeling simulation result protrudes into the binding pocket, thereby hindering subsequent docking simulations. This residue was corrected based on a known crystal structure of a different enzyme (yellow; PDB ID: 3LV2) with the correct Ile orientation at the same position. [file 12934_2016_566_MOESM2_ESM.tif]

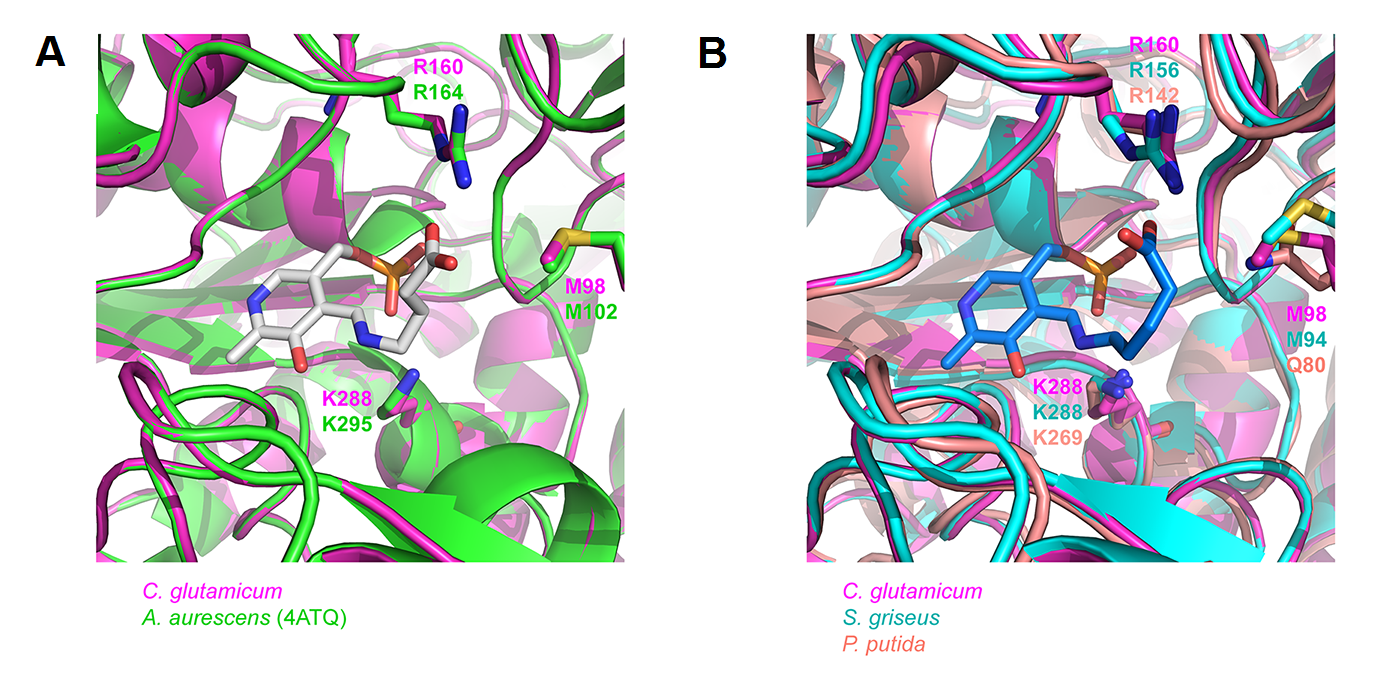

Supplement: Supplementary file 3 — 10.1186/s12934-016-0566-8 Molecular docking of external aldimine with C. glutamicum GabT. (A) Molecular docking of external aldimine (grey; PLP and γ-aminobutyrate) to homology-modeled GabT from C. glutamicum (magenta). A known crystal structure of GabT from Arthrobacter aurescens (green, PDB ID: 4ATQ) (Bruce et al. [49]) is superimposed for comparison. (B) Molecular docking of external aldimine (blue; PLP and AVA) to homology-modeled GabT from C. glutamicum (magenta). Homology-modeled DavT from P. putida (salmon) and homology-modeled GabT from S. griseus (cyan) are superimposed for comparison. [file 12934_2016_566_MOESM3_ESM.tif]

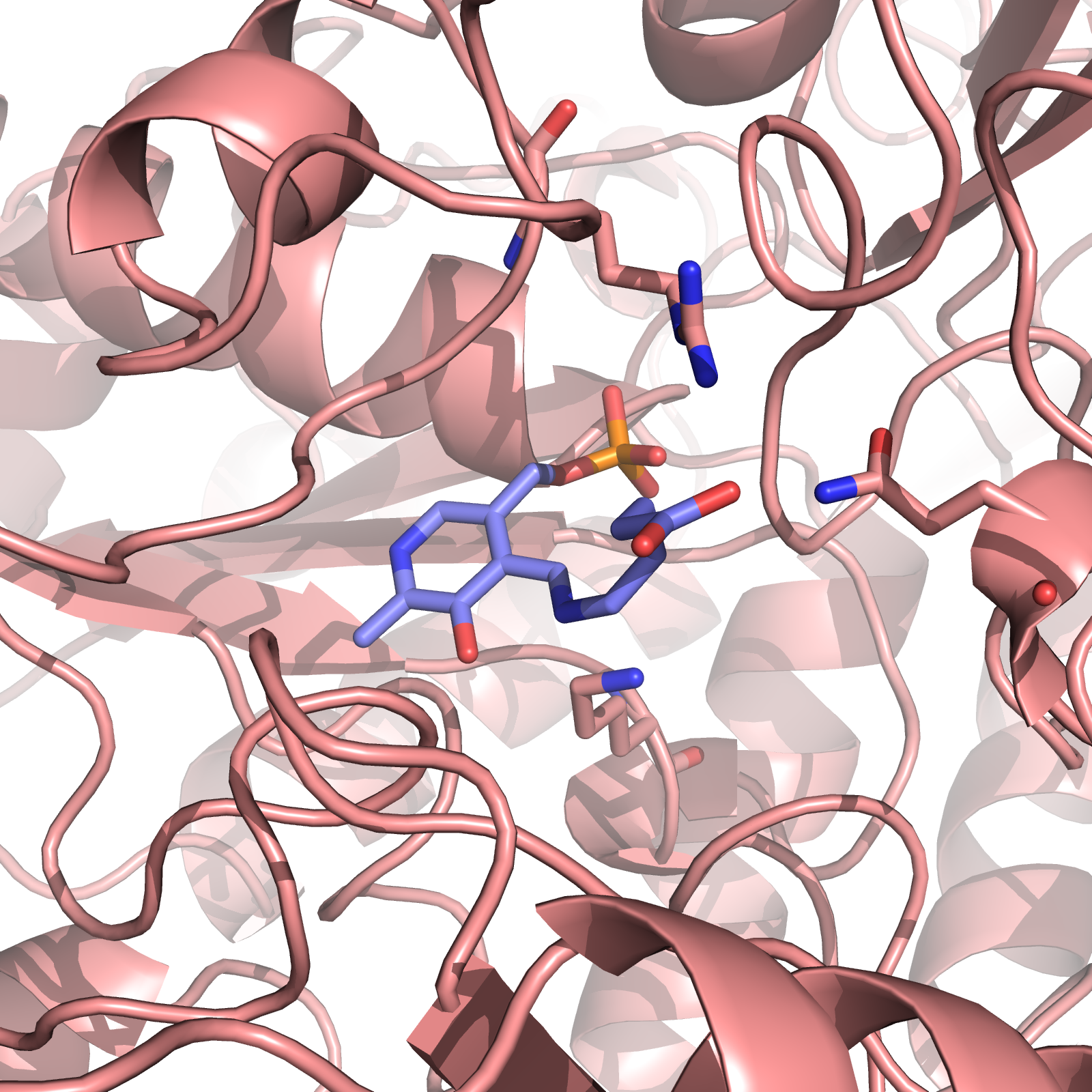

Supplement: Supplementary file 4 — 10.1186/s12934-016-0566-8 Simulation of molecular docking of 5AVA to homology-modeled DavT from P. putida KT2440. DavT was homology-modeled based on a known structure (PDB ID: 1SFF). [file 12934_2016_566_MOESM4_ESM.tif]
