# Supplementary material for: Metabolic engineering of Corynebacterium glutamicum for enhanced production of 5-aminovaleric acid
Source: Microb Cell Fact. 2016 Oct 7;15:174. doi: 10.1186/s12934-016-0566-8 (PMC5054628; doi:10.1186/s12934-016-0566-8)
Supplement: Supplementary file 5 — 10.1186/s12934-016-0566-8 Strains and plasmids used in this study. [file 12934_2016_566_MOESM5_ESM.docx]

**Table S1** Strains and plasmids used in this study.

| Strain/Plasmid | Description | Source or Ref |  |
| --- | --- | --- | --- |
| ***E. coli* strains** |  |  |  |
| TOP10 | General cloning purpose. Genotype: F^-^ *mcrA* Δ( *mrr-hsd*RMS-*mcr*BC) Φ80*lac*ZΔM15 Δ *lac*X74 *rec*A1*ara*D139 Δ( *araleu*)7697 *gal*U *gal*K*rps*L (StrR) *end*A1 *nup*G | Invitrogen |  |
| DH5*α* | General cloning purpose. Genotype: F^-^ Φ80*lac*ZΔM15 Δ(*lac*ZYA-*arg*F) U169 *rec*A1 *end*A1 *hsd*R17(r_k_^-^, m_k_^+^) *pho*A *sup*E44 *thi*-1*gyr*A96 *rel*A1 λ^-^ | Invitrogen |  |
| JM110 | Producing DNA plasmids free of Dam or Dcm methylation. rpsL (Strr) thr leu thi-1 lacY galK galT ara tonA tsx dam dcm supE44 Δ(lac-proAB) [F' traD36 proAB lacIqZΔM15] | Stratagene |  |
| S17-1 | RP4 derivative integrated in the chromosome for providing transfer functions. | ([Schafer et al., 1994](#_ENREF_55)) |  |
| ***C. glutamicum* strains** |  |  |  |
| BE | KCTC 12390BP, Expired industrial L-lysine producer strain from a local company | Daesang Corp, Korea |  |
| AVA2 | BE *ΔgabT* | This study |  |
| **Plasmids** |  |  | |
| pEKEx1 | *E*. *coli*-*C*. *glutamicum* shuttle vector, Km^R^, P_tac_, *lacI^Q^* | ([Eikmanns et al., 1991](#_ENREF_21)) | |
| pKE112DavAB | pKE112-MCS derivative, Ap^R^, *P*. *putida* ATCC12633 *davAB* | ([Park et al., 2013b](#_ENREF_45)). | |
| pKCA212-MCS | An in-house *E*. *coli*-*C*. *glutamicum* shuttle vector with pCC1 origin and p15A origin, Km^R^ | This study | |
| pKCA212davAB | pKCA212-MCS and pKE112-DavAB derivative, Km^R^, P_tac_, *P*. *putida* ATCC 12633 *davAB* | This study | |
| pJS30 | pEKEx1 derivative, *P*. *putida* ATCC 12633 *davA*, *P*. *putida* ATCC 12633 *davB* | This study | |
| pJS38 | pEKEx1 derivative, codon-optimized *davA*, codon-optimized *davB* | This study | |
| pJS57 | pEKEx1 derivative, P_Sod_, *ΔlacI*, *Δ*P_tac_ | This study | |
| pJS58 | pEKEx1 derivative, P_Tuf_, *ΔlacI*, *Δ*P_tac_ | This study | |
| pJS59 | pJS57 derivative, codon-optimized *davA*, codon-optimized *davB* | This study | |
| pJS60 | pJS58 derivative, codon-optimized *davA*, codon-optimized *davB* | This study | |
| pJS113 *beta* | pK19mobSacB derivative, *gabT* deletion vector. Designed to delete 330 nt in-frame. | This study | |
| pCES208 | *E*. *coli*-*C*. *glutamicum* shuttle vector, Km^R^, P_tac_ | Park et al., 2008 | |
| pCES208H36GFP | pCES208 derivative, P_H36_, eGFP, Km^R^, | Yim et al., 2013 | |
| p36davAB1 | pCES208 derivative, P_H36_, codon-optimized *davA*, codon-optimized *davB* | This study | |
| p36davAB2 | pEKEx1 derivative, P_H36_, codon-optimized *davA*, codon-optimized *davB* | This study | |
| p36davAB3 | pCES208 derivative, P_H36_, His-tagged and codon-optimized *davA*, codon-optimized *davB* | This study | |
|  |  |  | |
