# Supplementary material for: Metabolic engineering of Corynebacterium glutamicum for enhanced production of 5-aminovaleric acid
Source: Microb Cell Fact. 2016 Oct 7;15:174. doi: 10.1186/s12934-016-0566-8 (PMC5054628; doi:10.1186/s12934-016-0566-8)
Supplement: Supplementary file 6 — 10.1186/s12934-016-0566-8 Primers used in this study. [file 12934_2016_566_MOESM6_ESM.docx]

**Table S2** Primers used in this study. The restriction enzyme sites are underlined. Additional RBS sequence for expression of the following gene is indicated by boldface.

| Name | Sequence (5’-3’) | Purpose |
| --- | --- | --- |
| Psod F EcoRV | GTGTGATATCCCATAACAGGAATGTTCCTT | pJS57 |
| Psod R EcoRI | AGGTGAATTCGGGTAAAAAATCCTTTCGTA | pJS57 |
| Ptuf F EcoRV v2 | TTGTTAGATATCTTGAAATC | pJS58 |
| Ptuf R EcoRI | AATCGAATTCTGTATGTCCTCCTGGACTTC | pJS58 |
| 113 i1F beta | ATGCCTGCAGGGAGATGGCGATGGAGTTAAGCCGTT | pJS113 *beta* |
| 113 i1R beta | ACGGAGATCGCGCGCTCTGCGGTGAAGCGGGCAGCTGCGG | pJS113 *beta* |
| 113 i2F beta | CCGCAGCTGCCCGCTTCACCGCAGAGCGCGCGATCTCCGT | pJS113 *beta* |
| 113 i2R beta | CGACCTGCAGTCGGTGAGTGCTTCGAAGATTGC | pJS113 *beta* |
| JW01A-F | TGGATCCATGCATCACCATCACCATCATCGCATCGCACTGTACCA | p36davAB1 |
| JW01A-R | TAATGGCCCCCGAGGCCTTAGCCTTTACGCAGGTGCA | p36davAB1, p36davAB3 |
| JW01B-F | ATAGCATGCGGCCGC**AAAGGAGGAAAATC**ATGAACAAGAAGAATCGACACCCC | p36davAB1, p36davAB3 |
| JW01B-R | CACGAATGCGGCCGCTTAATCTGCCAGGGCGATCG | p36davAB1 |
| JW02H-F | AGATATCGGTACCTCTATCTGGTGCCC | p36davAB2 |
| JW02H-R | TGAATTCGGATCCCATGCTACTCCTAC | p36davAB2 |
| JW02AB-F | TGAATTCATGCATCACCATCACCATCATCGCATCGCACTGTACCA | p36davAB2 |
| JW02AB-R | CACGAATCTGCAGTTAATCTGCCAGGGCGATCG | p36davAB2 |
| JW03A-F | TGGATCCATGCATCACCATCACCATCATCATCGCATCGCACTGTACCA | p36davAB3 |
| JW03B-R | CACGAATGCGGCCGCTTAATGATGGTGATGGTGATGATCTGCCAGGGCGATCG | p36davAB3 |
