# Supplementary material for: Metabolic engineering of Corynebacterium glutamicum for enhanced production of 5-aminovaleric acid
Source: Microb Cell Fact. 2016 Oct 7;15:174. doi: 10.1186/s12934-016-0566-8 (PMC5054628; doi:10.1186/s12934-016-0566-8)
Supplement: Supplementary file 7 — 10.1186/s12934-016-0566-8 Sequence of codon optimized davA and davB genes from P. putida. [file 12934_2016_566_MOESM7_ESM.docx]

**Table S3** Sequence of codon optimized *davA* and *davB* genes from *P. putida.*

| Gene | Codon optimized sequence |
| --- | --- |
| *davA* | atgcgcatcgcactgtaccaaggcgcacccaagccactagacgttcctggtaaccttcaacggctgcgccaccaggcgcagctggcagctgaacgcggagctcagttgctggtgtgcccagagatgttcctcaccggctacaacattggcctggcccaagtcgaacgtctcgccgaagccgcagatggcccagcagcaatgaccgtggtcgaaatcgctcaggctcaccgcatcgcaattgtttacggttacccggagcgcggtgatgacggagctatctacaactccgttcagttgatcgatgcgcatggacgatctctgtcaaattatcgcaagacgcacttgttcggtgaactcgatcgctcgatgttctcccctggtgcggaccacttcccagtcgtggaactggaaggctggaaggttggacttcttatctgttacgacatcgagttcccagagaacgcccgtcgactagcgttggatggagccgagcttatccttgtgcccaccgctaacatgactccgtacgattttacctgccaagtgactgtccgtgcgagggcacaggaaaatcagtgctacctcgtatatgcaaactactgcggtgctgaagacgagattgaatattgtgggcaatctagcattattggaccggatggctccttgctcgctatggccggtcgcgatgaatgccagttgcttgcagagcttgagcatgagcgggtcgttcaggggcgtacagcttttccttatttaaccgacctccgtcaggagctgcacctgcgtaaaggctaa |
| *davB* | atgaacaagaagaatcgacaccccgccgacggcaagaagccgattaccattttcggaccagatttcccttttgctttcgatgattggctagaacacccagcaggcctgggaagcattccagctgagcgccatggagaagaggtggctatcgtcggagctggtatcgctggcctcgtagcggcatacgagctgatgaagctgggcctcaagcctgtggtgtatgaggcttccaagctcggcggccggctccgctcccaagccttcaatggaactgacgggatcgttgccgagctgggtggcatgcgcttcccagtgtcttccactgccttctaccactacgtcgacaaattgggcctggaaacgaaacccttccccaatcctttgaccccagcttccggaagtacggttattgatcttgaaggacagacctattacgccgagaaacctacagaccttccacaactgtttcatgaggttgccgacgcatgggctgatgctctggagtcgggtgcgcagttcgccgatatccagcaggcaatccgcgatcgtgatgtaccacgccttaaggaattatggaacaagttggttccactgtgggacgaccgtaccttctacgacttcgtcgctacctctcgctcctttgctaaactgagctttcaacacagagaagtgtttggccaggtcggtttcggcaccggcggttgggattcggacttccctaacagtatgttggaaatcttccgcgtggttatgaccaactgcgacgaccaccagcacctggttgttgggggtgtggaacaagtcccacaaggaatctggcgccacgtgccggaacgttgtgtgcattggccagaagggactagcctgagcacgctgcatggtggcgcaccgcgtaccggtgtcaagcgcattgcccgcgcatccgatggccgcttggcagtcacggacaactggggtgatacccgccactattccgcagtactagctacctgtcagacatggttgcttaccactcaaatcgactgcgaagaatctctgttctcgcaaaagatgtggatggcactggaccggacccgctacatgcagtcgtctaaaacctttgtcatggtcgacaggccgttctggaaggataaggaccctgagaccggtcgtgacctgctgagcatgaccctcactgatcgtctcactcgcggcacttatctttttgataacggtaacgataaacccggggtgatctgcctgtcatactcatggatgtctgatgcgctgaagatgctgccacacccggtggagaagcgcgtacagcttgccctggatgcgctcaagaagatttatccgaaaaccgatatcgcaggccatatcatcggcgatccaatcacggtttcctgggaggccgacccctactttctcggcgcgttcaaaggcgcgttaccgggtcattaccgctacaaccagcgaatgtacgcgcacttcatgcagcaggatatgccggcagagcagcgcggtatttttattgctggtgatgacgtgtcatggacccctgcctgggttgaaggcgcggtccagacatctctgaacgcagtgtggggtatcatgaatcactttggtgggcacacccacccagacaatccaggcccgggagatgtgttcaacgagatcggcccgatcgccctggcagattaa |
